# The Tree Theme Method^®^ (TTM), an occupational therapy intervention for treating depression and anxiety: study protocol of a randomized controlled trial

**DOI:** 10.1186/s40359-015-0097-9

**Published:** 2015-11-09

**Authors:** A. Birgitta Gunnarsson, Petra Wagman, Carita Håkansson, Katarina Hedin

**Affiliations:** Department of Research and Development, Region Kronoberg, PO Box 1223, SE-351 12 Växjö, Sweden; Department of Clinical Neuroscience and Rehabilitation, The Sahlgrenska Academy at University of Gothenburg, Gothenburg, Sweden; Department of Rehabilitation, School of Health and Welfare, Jönköping University, Jönköping, Sweden; Division of Occupational and Environmental Medicine, Lund University, Lund, Sweden; Department of Clinical Sciences in Malmö, Family Medicine Lund University, Lund, Sweden

**Keywords:** Adults, Affective disorders, Art therapy, Everyday activities, Mental health, Randomized control trial

## Abstract

**Background:**

Depression and anxiety disorders are increasing among the general population in the Western world. Individuals may need several kinds of treatment in order to maintain health, such as cognitive behavioural therapy (CBT) and drug treatment. However, having an everyday life that “works” is also important, suggesting a need for interventions based on activities that facilitate a satisfying everyday life. There is still lack of such evidence-based interventions. The Tree Theme Method^®^ (TTM) is an occupational therapy intervention designed for a client-centred context in which an individual develops strategies to become an actor in his or her everyday life. Previous studies of the TTM have focused on process evaluation; therefore, further studies are needed to evaluate the method’s effects. The aim of this paper is to outline an intervention that can evaluate the effects of the TTM in terms of psychological symptoms, as well as everyday occupations and well-being, in patients suffering from depression and anxiety.

**Methods/Design:**

This randomized clinical trial includes patients from three Swedish counties randomized to either intervention or treatment as usual. Men and women aged 18–65 years who have been diagnosed with either depression or anxiety are eligible for inclusion. Data collection is carried out at baseline, and outcomes are assessed at the end of intervention, as well as at 3 months and 12 months after intervention ends. The outcomes measured are psychological symptoms, everyday activities, and health-related factors.

**Discussion:**

Depression and anxiety may create difficulties for individuals in the activities of their everyday lives to the extent that they require diagnosis and intervention. Despite this reality, evidence-based interventions that focus on everyday activities are lacking. Therefore, it would be useful to design a specific method for occupational therapy intervention that does precisely that. This study provides insight into the effects of the TTM, comparing it to occupational therapy treatment as usual.

**Trial registration:**

ClinicalTrials.gov: NCT01980381; registered November 2013.

## Background

Depression and anxiety disorders are two major diseases, and their prevalence is increasing among the general population in the Western world. In Sweden, about 15 % of men and 25 % of women are affected at some point in life to such an extent that they need treatment [1]. When an individual suffers from depression or anxiety disorders, his or her ability to cope with various activities in everyday life [2, 3] and to relate to others may be limited, while experiencing various psychological symptoms and possible side effects from drugs. The problems related to the individual’s disease also contribute to high sickness rates. In 2011, depression and anxiety disorders each accounted for 12 % of the sicknesses in Sweden [4].

**Fig. 1:**
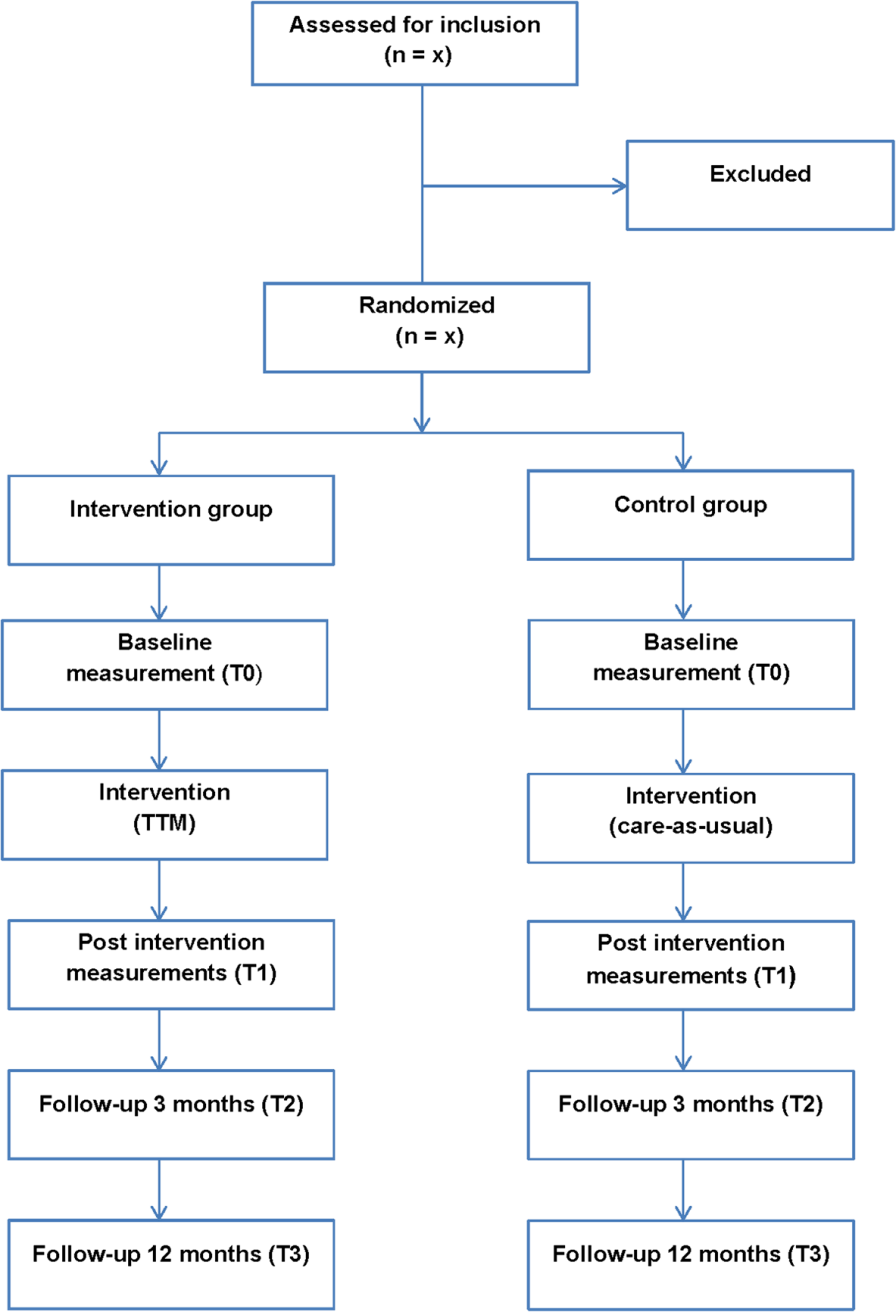
Overview of study procedure

The Swedish National Board of Health and Welfare [1] recommends psychological approaches, such as cognitive behavioural therapy (CBT) and drug treatment, for patients diagnosed with depression or anxiety. But the Swedish National Board of Health and Welfare also suggests other interventions that are adapted to the individual’s needs, and the board has stated that having a satisfying everyday life at home and at work or school, along with a sense of belonging in a social context, is just as important as obtaining a proper diagnosis and appropriate psychological or pharmacological treatment [1]. We have not found any evidence-based treatment interventions that focus on everyday life, such as activities in connection with self-care, productivity (at home, at work, of at school), leisure, or relationships to others.

The Tree Theme Method^®^ (TTM) is an intervention that can complement CBT and pharmacological treatment. The TTM is designed for a client-centred clinical occupational therapy context [5] in which the patient paints pictures of trees that represent certain periods in his or her life. These paintings are used as a starting point for the patient to tell his or her life story, focusing on the mix of activities and routines in everyday life [6]. The intervention is based on knowledge about art therapy [7], life storytelling [8, 9] and the value of meaningful activities [10] in maintaining good health. The TTM intervention involves five sessions and is carried out during a defined period of 6–9 weeks. The approach aims to increase the individual’s ability to cope with everyday life [5], and its intention is to develop strategies for becoming active, thus enhancing patients’ satisfaction with the mix of activities and routines that compose their everyday lives [5].

The TTM has been evaluated in terms of processes. The first study to describe the method presented the case of a woman diagnosed with depression and anxiety who, after taking part in the intervention, became an actor in her own life – that is, she went from being unable to care for her family or manage her work to being active and effective in her everyday routine [5]. In an additional study [11], the TTM was evaluated in a group of patients (6 men and 29 women) diagnosed with depression or anxiety disorders, this time examining changes in their perceptions of health and activity after completing the intervention. The results showed consistently positive outcomes for the patient group regarding psychological symptoms, well-being, activity performance, therapeutic alliance, and patient satisfaction with the intervention. Further, better outcomes and higher client satisfaction correlated with higher ratings of the therapeutic alliance [11]. The TTM has also been evaluated in qualitative studies focusing on patients’ and therapists’ experiences of the method, including the therapeutic alliance [12, 13]. Overall, patients reported feeling that the TTM suggested that they could become focused and expressive, and that this in turn meant that they could change their everyday lives. They argued that the TTM marked a turning point at which they gained new perspectives on self-care, productivity, leisure, and their relationships with others [13]. The therapists experienced the TTM as a structured method for intervention, and they were impressed with the fact that the method could initiate a process of significant change for patients in such a short time (i.e., five sessions) [12]. A 3-year follow-up [14] used the same outcome measures presented in Gunnarsson and Eklund [16] – psychological symptoms, well-being, and activity performance – and demonstrated sustainable positive outcomes, particularly regarding increased ability to perform everyday activities both at home and at work.

It is important to offer patients with depression and anxiety disorders various interventions other than CBT and drug treatment alone, such as therapy that aims to enhance their satisfaction their everyday routines, as the TTM does. The focus of previous studies examining the TTM has been process evaluation, and therefore further studies should evaluate this intervention’s effects.

## Objectives

This paper outlines an occupational therapy intervention in order to evaluate the effects of the TTM. The primary objective concerns the psychological symptoms of depression and anxiety in diagnosed patients, as well as patients’ perceptions of their own performance of everyday activities and their satisfaction with that performance. The secondary objective is to evaluate various health-related factors, intervention-related aspects, and health-care consumption. We compare the outcomes for participants in the intervention group (TTM) with those in the control group (treatment as usual = occupational therapy as usual) in order to identify and explore the effects of TTM.

## Methods

### Study design

The study is a randomized controlled clinical trial (RCT) [15] of nonpharmacological treatment according to CONSORT recommendations [16]. Participants are randomized to either an intervention group or a control group. The study design is shown graphically in Fig. 1.

### Participants

Our study sample includes men and women between 18 and 65 years old who have been diagnosed by a physician with depression and/or anxiety and have been assessed as encountering difficulties in their everyday activities. We exclude patients with severe somatic disease or psychosis, as well as those with language difficulties or cognitive disorders that would impede their understanding of the self-rating questionnaires.

### Context and procedure

This study is being conducted at primary health-care centres and general outpatient mental-health-care units in three counties in the south of Sweden. Thirteen occupational therapists specifically trained in the TTM intervention ask patients who meet the inclusion criteria to participate and obtain informed consent from those who agree. In order to assess the therapeutic alliance, the occupational therapist does not meet a patient more than twice before asking him or her to participate in the study. Patients who do not wish to participate in the study receive the usual treatment. After recruiting, each patient is allocated to either the TTM or the control group (occupational therapy as usual). Drug treatment continues as prescribed, and dropouts are documented during the study. The same occupational therapists perform both TTM intervention and treatment as usual. In order to ensure that the occupational therapists actually deliver two different treatments they complete fidelity forms after each session.

### Intervention

#### The TTM intervention

Participants who are randomized to the TTM complete five sessions of 60 min each during a period of 6–9 weeks. In the TTM, each of the first four sessions starts with a progressive relaxation in which a specific theme is introduced, and the patient paints a symbolic tree. This picture is then used as a starting point for patients to tell their life stories, focusing on everyday activities. This part of the intervention constitutes a reflective dialogue between the patient and the occupational therapist aimed at identifying necessary changes in the patient’s life. The theme of the tree differs from one session to the next, and each tree represents a particular period of life: the present, childhood, adolescence, adulthood. In the fifth session, the focus is on story making and on shaping plans for the future. Patient and therapist also decide on tasks, linked to the patient’s identified difficulties and needs, that the patient completes between sessions.

#### Treatment as usual

Participants who are randomized to the control group complete five sessions of 60 min each during a period of 6–9 weeks. Each occupational therapist has defined what is meant by *occupational therapy as usual*. The focus of these sessions is dialogue, sometimes in combination with activities, regarding the patient’s well-being and his or her current activities in everyday life. Patients may or may not be assigned tasks between sessions.

### Training occupational therapists in the TTM

The occupational therapists that participated in this intervention study completed 50 h of standard training in the frames and techniques of the TTM provided by the Swedish Association of Occupational Therapists involving theory about client-centred occupational therapy, art therapy, and occupational storytelling and story-making with a focus on everyday activities. The training also involved practical exercises, including painting the different tree themes, in which the therapists work in role playing pairs, switching between client and therapist. In addition, the occupational therapists in this study also completed two additional days of training the study’s objectives and implementation.

### Data collection

Data collection for this study is being carried out by project assistants on four occasions: 1 to 2 weeks before intervention, immediately after intervention, 3 months after completion, and 12 months after completion. Sociodemographic variables—namely, gender, age, diagnosis, accommodation, social networks, level of education, and employment—are documented. In addition, physical activity and health-care consumption (number of visits to service providers in the preceding month), inpatient care, pharmacological treatment (for insomnia, depression, or anxiety), and pharmacological-treatment compliance are recorded, along with any other health-care consumption. The data collection is blinded, i.e. the project assistants are unaware of treatment assignment.

### Primary outcomes

*Psychological symptoms* are measured with the Symptom Checklist-90-R (SCL-90-R) [17]. This self-rating questionnaire measures psychological problems; it consists of 90 items rated on a five-step categorical Likert scale from zero to four, with higher scores indicating greater severity. In this study, the Global Symptom Index (GSI) of SCL-90-R is calculated. The SCL-90-R is used frequently and has been found to be reliable and valid [17, 18].

*Depression* is measured with the Montgomery-Åsberg Depression Rating Scale (MADRS-S) [19]. The self-rating questionnaire consists of nine items, each rated on a seven-step Likert scale from zero to six, with higher scores indicating greater severity. The MADRS-S is a commonly used measurement in clinical practice; it has been shown to have good internal consistency [20] and to be a useful screening instrument for depression [21].

*Anxiety and depression* are measured with the Hospital Anxiety and Depression Scale (HADS) [22]. The self-rating questionnaire comprises 14 items, and two subscales measure anxiety (7 items) and depressive symptoms (7 items), each rated on a four-step categorical Likert scale from zero to three, where higher scores indicate greater severity. The HADS is commonly used in clinical practice and has performed well in assessing symptoms of depression and anxiety disorders in a general population [22, 23].

*Everyday activities* are measured from three aspects: performance of and satisfaction with the performance of activities, satisfaction with everyday activities, and balance in everyday activities. *Performance of and satisfaction with the performance of activities* are measured with the Canadian Occupational Performance Measurement (COPM) [24], a semi-structured instrument focusing on self-care, productivity, and leisure. The patient identifies problematic tasks and activities and then rates his or her actual performance and satisfaction with that performance. The ratings apply a ten-step numeric Likert scale from 1 (not important at all) to 10 (extremely important). The patient then rates his or her performance and satisfaction with the performance of targeted tasks. The Swedish version used here has demonstrated high responsiveness to change [25] and clinical utility [26].

*Satisfaction with everyday activities* is measured with the tool Satisfaction with Daily Occupations (SDO) [9], a semi-structured interview focusing on perceived satisfaction with everyday activities in work, leisure, domestic tasks, and self-care. The individual first indicates whether he or she presently performs an activity and then rates his or her satisfaction with that performance on a seven-step numeric Likert scale from 1 (worst possible) to 7 (best possible). The original version has nine items and has demonstrated satisfactory internal consistency, construct validity [27, 28], and test-retest reliability [28]. The present study used a 14-item version in order to cover more activities in the areas of leisure, domestic tasks, and self-care; this version has also shown satisfactory internal consistency [29].

*Balance in everyday life activities* is measured with the Occupational Balance Questionnaire (OBQ) [30], a 13-item self-rating questionnaire that indicates an individual’s perception of balance. Balance is rated on a six-step categorical Likert scale, from 0 (completely disagree) to 5 (completely agree). The OBQ has shown good internal consistency and test-retest reliability [30].

### Secondary outcomes

*Health-related aspects* are measured from three perspectives: sense of coherence, experience of control, and quality of life. *Sense of coherence* is measured with the Sense of Coherence (SOC) [31] tool, a self-rating questionnaire of 13 items that indicates how well an individual copes with stress and stays healthy. Items are rated on a seven-step numeric Likert scale from 1 (very often) to 7 (very seldom or never). The Swedish 13-item SOC used here is a common measurement that has demonstrated good test-retest reliability and internal consistency [32].

*Experience of control* is measured with the Mastery Scale [33]. This self-rating questionnaire comprises seven items and indicates the extent to which an individual experiences control and mastery of situations that influence his or her everyday life. Items are rated on a four-step categorical Likert scale from 1 (completely agree) to 4 (do not agree at all). This questionnaire has been found to have good internal consistency and construct validity [34].

*Quality of life* is measured with the Manchester Short Assessment of quality of life (MANSA) [35], an interview-based questionnaire of 12 items, including the individual’s general rating of life satisfaction and his or her satisfaction with work, finances, social relationships, leisure, housing situation, family relationships, and both physical and mental health. Items are rated on a seven-step numerical Likert scale from one to seven, with higher scoring reflecting greater satisfaction. The Swedish version used here has been found reliable in terms of internal consistency and construct validity [36].

*Intervention-related aspects* are measured by two factors: therapeutic alliance and patient satisfaction. *Therapeutic relationship* was measured using the Helping Alliance Questionnaire (HAq-II) [37], which indicates the strength of the alliance between patient and therapist. There is both a patient version and a therapist version, allowing each to independently rate the therapeutic alliance. The HAq-II consists of 19 items scored on a six-step categorical Likert scale from 1 (strongly disagree) to 6 (strongly agree). The questionnaire has been found internally consistent and reliable for test-retest purposes [37], and it demonstrates good convergent validity [37, 38].

*Patient satisfaction* is measured with the Client Satisfaction Questionnaire (CSQ) [39], which indicates the patient’s satisfaction with the received intervention. The eight-item instrument uses a four-step categorical Likert scale from 1 (very dissatisfied) to 4 (very satisfied), and it has been found to have good internal consistency [40] and construct validity [41, 42].

### Sample size/power calculation and randomization

The power calculation indicates that 60 patients would be needed in each group to obtain an effect size of 3.6 (scale on 9–63) for the outcome variable Satisfaction with Daily Occupations (SDO), with 80 % power (*p* = 0.05). To compensate for possible losses, a total of 130 patients will be recruited. After granting informed consent, patients are allocated (by means of sealed envelopes prepared by a researcher not involved in the study) to either intervention or treatment as usual. In all, 65 patients will be randomized to the TTM (intervention group) and 65 to treatment as usual (control group).

### Statistical analysis

At the study’s completion, data analysis will be performed on the principle of intention to treat [15]; in other words, outcomes will be analysed using the basis of all participants. Missing data for dropouts will be estimated using the last data obtained before a participant dropped out. Descriptive and analytic statistics will be used to compare the outcomes between the intervention group and the control group at baseline, after intervention, and at 3- and 12-month follow-ups. As all variables will be assessed on either a nominal or an ordinal scale, statistical analysis with non-parametric methods will be used.

### Ethical issues

The study has been approved by the Regional Ethical Review Board in Linköping (Dnr 2012/232-31) and is being conducted in line with the Helsinki Declaration [43]. All participants receive verbal and written information about the aims and procedures of the study, and each provides his or her informed consent in writing. Concerning confidentiality, participants’ anonymity and integrity are guaranteed, and data are kept locked so that no unauthorized person has access to them. All data will be analysed at the group level.

### Time frame of the study

The inclusion process began in January 2013 and is scheduled to end in June 2016. The intervention for each patient starts soon after his or her inclusion, and all data collection will have been completed 1 year after the last-included patient’s intervention has ended.

## Discussion

Depression and anxiety disorders are common and may lead to difficulty functioning in everyday life, at home as well as at work. Although these illnesses may affect people’s daily lives to the extent that they require medical care and treatment, currently no evidence-based interventions focus on activities of self-care, productivity, leisure, and relationships with others. Therefore, there is a need for this study, which tests the design of a specific occupational therapy intervention method that concentrates on everyday activities. This study, once completed, will provide insight into the effects of the TTM compared to occupational therapy as usual. If it turns out that the TTM is effective, the method can be implemented more frequently.

On the other hand, if we find no differences in outcomes between the intervention group and the control group, this may support the theory that outcomes are influenced by the therapeutic alliance. In previous research examining the TTM [11], the therapeutic alliance has proved important to outcomes in terms of psychological symptoms, well-being, activity performance, and patient satisfaction with the intervention—a finding that aligns with several reviews. For instance, the importance of the therapeutic alliance to treatment outcomes has been identified in treating eating disorders [44], borderline personality disorders [45], and cancer [46]. Therefore, we decided to have the same occupational therapists carry out both the TTM intervention and the occupational therapy treatment as usual.

## Abbreviations

HADS: Hospital anxiety and depression scale
COPM: Canadian occupational performance measurement
CSQ: Client satisfaction questionnaire
CBT: Cognitive behavioural therapy
CONSORT: Consolidated standards of reporting trials
GSI: Global symptom index
HAq-II: Helping alliance questionnaire
MANSA: Manchester short assessment of quality of life
MADRS-S: Montgomery-Åsberg depression rating scale
OBQ: Occupational balance questionnaire
RCT: Randomized controlled trial
SDO: Satisfaction with daily occupations
SOC: Sense of coherence
SCL-90-R: Symptom checklist-90-R
TTM: Tree Theme Method^®^
